# 

**Published:** 1877-05-20

**Authors:** 


☞ All communications relating to the business of THE RECORD, for the year 1877, must be addressed to
DR. R. C. WORD,
Business Manager, Southern Med. Rec.
Atlanta, Ga.

☞ Brief and practical communications are solicited on all subjects pertaining to medicine, also reports of cases in practice.

☞ Send money by check, postal order, or registered letter.

☞ Write your name, post-office, county and State plainly.

☞ See advertisement of *Medical Register and Directory.*

COLLABORATORS AND CONTRIBUTORS.

L. B. Anderson, M.D., Va.
Swan M. Burnett, M.D., D. C
J. M. Bristow M.D., Texas
J. W. Booth, M.D., N. C.
W. F. Barr, M.D., Va.
B. W. Corn, M.D., Texas.
John Castor, M.D., Iowa.
E. T. Easley. A.M., M.D., Ark.
A. H. Goelet, M.D., N. Y.
E. C. Gehrung, M.D., Mo.
G. D. Hodge, M.D., Ark.
S. M. Hogan, M.D., Ala.
A. McLane Hamilton, M.D., N.Y.
C. C. Vanderbeck, M.D., Pa.
Z. B. Herndon, M.D., Va.
Lindsav Johnson, M.D., Ga.
A. R. Kilpatrick, M.D., Tex.
R. Kells, M.D., Miss.
W. L. Lipscomb, M.D., Miss.
J. M. Lewis, M.D., Miss.
G. A. Dyer, M.D., Ind.
C. H. Lathrop, M.D., Iowa.
A. A. Lyon, M.D., La.
M. Lewis, M.D., Tenn.
G. M. Rivers, M.D., S. C.
A. S. Payne, M.D., Va.
A. M. Whitehead, M.D., O.

